# Electric fields in a counterflow nonpremixed flame: measurement and simulation

**DOI:** 10.1038/s41598-023-34769-6

**Published:** 2023-05-10

**Authors:** Jin Park, Jinwoo Son, Thomas D. Butterworth, Min Suk Cha

**Affiliations:** 1 Physical Science and Engineering Division (PSE), CCRC, King Abdullah University of Science and Technology (KAUST), Thuwal, 23955 Saudi Arabia; 2 Brightsite Plasmalab, Circular Chemical Engineering, Maastricht University, Brightlands Chemelot Campus, Urmonderbaan 22, Geleen, 6167 RD The Netherlands

**Keywords:** Mechanical engineering, Plasma physics

## Abstract

In electric field modified flames, the electric body force on fluid elements can play a role in modifying the flow field, affecting flame characteristics by this modified flow motion. Numerical studies have developed ion kinetic mechanisms and appropriate transport models for charged species, validating them with a voltage-current trend in 1D premixed flames. Recent experimental approaches have measured the electric field by adopting the Electric Field Induced Second Harmonic generation (EFISH) technique. However, the quantification has turned out very challenging due to the inherent distortion in the EFISH signal, as well as inhomogeneous temperature and concentration fields in the combustion field. Here, we propose measurement and calibration schemes to quantify the EFISH signal in a laminar counterflow nonpremixed flame and present comparison with numerical results using an in-house multi-physics CFD (Computational Fluid Dynamics) code. Overall, the quantified electric fields agreed well with those from numerical simulation, specifically capturing null electric fields near the flame in the sub-saturated regime due to the electric field screening effect. In the saturated regime, notable discrepancy was found in a fuel stream when electrons moved through it: experiment indicated a significant number of negative ions in the fuel stream, whereas numerical results predicted negligible negative ions, due to the implemented ion-mechanism. This suggested that the experimentally obtained electric fields may serve as validation data for modeling studies to improve transport models and ion-mechanism. In-situ measurement of charged species in the presence of external electric fields should be a future work.

## Introduction

Flames—particularly those burning hydrocarbons—are known to show intrinsic electrical characteristics because charged particles such as electrons and ions are generated in a reaction zone via chemi-ionization^1^. The electrical aspects of flames and electrically modified flames have been investigated for almost a century. Lawton and Weinberg well developed and summarized early phenomenological findings and theoretical fundamentals behind^1^. Later on, ion-chemistry occurring in flames was extensively reviewed by Fialkov^2^. The first comprehensive ion reaction mechanism for hydrocarbon flames was configured by Prager et al*.*^3^, triggering following kinetic and numerical studies. Stimulated by advanced diagnostic and simulation capabilities, this topic has been revived since early 2000; those recent progresses were well reviewed in literature^4,5^.

An electric field, externally applied to a flame, induces a movement of charged particles by the Lorentz force acting on them, resulting primarily in an ionic current. As the applied electric field increases, the current increases quadratically (sub-saturated regime) and then becomes saturated being limited by the production rate of charged species (saturated regime)^6,7^. As a result of the ion current, a bulk flow motion (ionic wind) is generated by an electric body force due to a local electric field and the space charges in a fluid element^6,8–11^. Since flow characteristics significantly affect the characteristics of combustion and a flame via transport, stretch, and disturbance, the ionic wind may influence some phenomena of interest. For this reason, many studies have been conducted to utilize the electric fields to control propagating flame speeds^12^, reduce soot emissions^13^, and increase flame stabilities^14^. Recently, numerical studies with CFD (Computational Fluid Dynamics) modeling have advanced this research field^14^. Most previous studies only used one-dimensional current responses for a validation; thus, the numerical approaches can be further improved as other experimental data are available for comparison.

In a CFD simulation, to properly calculate the electric body force term to the momentum equation, the electric field and space charge distribution must be known. Therefore, the use of a proper ion-chemistry set and rigorous transport properties of charged species is central for the accurate estimation of the space charge distribution and, in turn, the electric field via a charged species transport equation and the Poisson equation (Gauss’s Law), respectively. A space charge profile is significantly affected by the mobilities and the production rate of the charged species. Thus, the electric field also includes the information of the ion-chemistry, the transport properties, as well as the externally applied electric field. This indicates that the electric field is not only important for the generation of the ionic wind but also useful for the comparison between experimental and numerical results, indirectly validating a used chemi-ionization mechanism and the transport modeling of the charged species in a numerical method.

Electric Field Induced Second Harmonic generation (EFISH) is a laser-based electric field measurement technique that has been recently employed in many plasma systems such as gas discharges^15–18^ and plasma jets^19^. The EFISH technique is known to have a good temporal and spatial resolution, because a short-pulsed high power laser system is usually used with a focused beam at a measuring point. Although there have been a few attempts to apply the EFISH method to combustion systems^20–23^, it has been difficult to calibrate the EFISH signal with a flame; this was partly due to problems determining third order susceptibility and local number density of species, since the EFISH signal depends on them. In addition, a recent study^24^ reported some limits in the EFISH method, emphasizing that the EFISH signal could be distorted by the length of the laser beam path exposed to the electric field^24^; specifically, the null electric field could not be measured if an incident beam was exposed to an electric field other than a measuring point. Therefore, EFISH measurement requires specific measuring and calibrating schemes to measure the electric field in a flame properly.

Toward this end, the scope of this work was to compare electric field data from an experiment and those from a numerical simulation, aiming to provide experimental data of reference and to discuss a predictive capability of the simulation. A canonical nonpremixed flame in a counterflow burner system was selected, since this has been a flame of interest in many previous studies^6,7,14,20,25^. To improve the measurement and calibration of EFISH proposed in the previous study^20^, (i) a ns-high-voltage switch was newly introduced to effectively freeze a space charge profile, and (ii) a new measurement scheme was developed to consistently maintain the integration length of the incident laser beam. A multi-physics CFD simulation, using an OpenFOAM based in-house code, was conducted for numerical electric field data. The state-of-the-art numerical models—chemi-ionization mechanism and transport data—from literatures^26–29^ were implemented to obtain up-to-date data based on the best available knowledge in the field.

## Experimental and numerical methods

### Experimental apparatus

**Figure 1:**
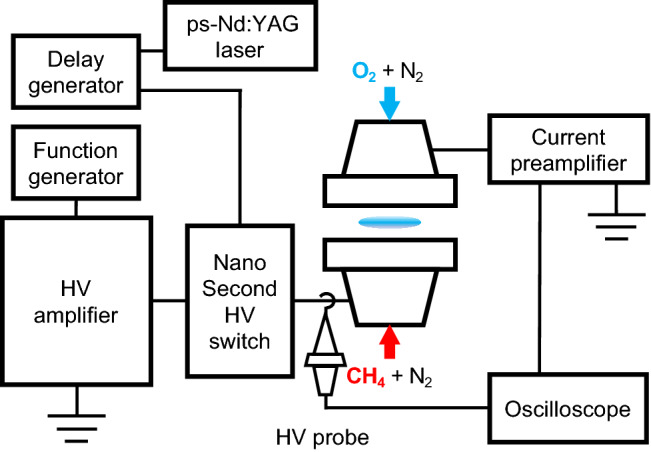
Schematic of experimental setup. Electrical configuration represents upward electric field with a positive HV (ETF: electron through fuel stream). HV was connected to the upper nozzle to apply downward electric field; lower nozzle was grounded (ETO: electron through oxygen stream).

The experimental apparatus consisted of a counterflow burner system, a power supply system, and a laser diagnostic system, as shown in Fig. 1. The same counterflow burner was used in our previous studies^8,20^. The central jet of the counterflow burner was supplied through a nozzle with an inner diameter of 10 mm; a coaxial nozzle (20-mm inner diameter) surrounded the central nozzle to supply N_2_ sheath flow. The central nozzle was machined in a tapered way, and thus the wall thickness between the central jet and the surrounding N_2_ sheath was negligible at the plane of exit. To apply a uniform external electric field in a gap between two opposed nozzles, a 1.5-mm thick perforated plate with a diameter of 80 mm was placed at the outlet of each nozzle. The diameter of a hole in the perforated plate was 0.8 mm, and the number density of the holes was 79/cm^2^. For electrical insulation, the upper and lower parts of the counterflow burner were placed on an acrylic frame, setting a separation distance between the opposed surfaces of the perforated plates at 10 mm. Cooling water controlled the temperature of the burner and consistently maintained the reactant temperature.

Methane, oxygen, and nitrogen established a non-premixed flame of interest. To locate the flame at a stagnation plane, the compositions of CH_4_/N_2_ and O_2_/N_2_ mixtures were controlled to satisfy the stoichiometric mixture fraction (*Z*_st_) to be 0.5, where $Z_{{{\text{st}}}} \, = \,{1}/\left\{ {{1}\, + \nu \left( {Y_{{{\text{CH}}_{{4}} }} /Y_{{{\text{O}}{}_{{2}}}} } \right)} \right\}$ with the stoichiometric mass ratio of O_2_ to CH_4_ (*ν*) and the mass fractions of CH_4_ ($Y_{{{\text{CH}}_{{4}} }}$) and O_2_ ($Y_{{{\text{O}}_{{2}} }}$) in the fuel and oxidizer stream, respectively^8^. The oxidizer stream was supplied from the upper nozzle, diluted with nitrogen (52.7% of O_2_ by volume); the methane was also diluted with nitrogen (22.2% of CH_4_ by volume) and supplied from the lower nozzle. Central jet velocities from both nozzles and the corresponding N_2_ sheath flow were set at 20 cm/s.

A power supply (Trek, PD07016) was used to apply the electric field to the flame. The power supply amplified a signal from a function generator (NF, WF1973) and applied direct current (DC) high-voltage (HV) to either the upper or the lower part of the burner, grounding the counter part of the burner. In this way, the direction of the electric field could be changed, leading electrons to move through either the fuel stream (ETF, Electron Through Fuel stream), or the oxidizer stream (ETO, Electron Through Oxygen stream). From this point on, the ETF and ETO indicated the direction of the electric field with respect to the flame configuration. The applied voltage (*V*_a_) and current were measured using an HV probe (﻿Tektronix, P6015A) and current preamplifier (Stanford Research System, SR570), respectively, with an oscilloscope (Tektronix, DPO7254C).

During the EFISH measurement, a ns-HV switch (BEHLKE, HTS 301-01-GSM) was employed to effectively freeze the motion of charged species in time and space. This switch (installed at the HV outlet of the power supply) rapidly lowered the applied voltage to zero in the order of 100 ns. Using the ns-HV switch to turn off the applied voltage, a space charge distribution, caused by the applied voltage, can be reasonably preserved during the voltage decay. This was confirmed by comparing the charge species profiles and the electric fields in extended simulations (see Figs. S1 and S2 in Supplementary Information, SI). A somewhat longer decaying time of the voltage was discovered using a negative applied voltage, therefore, the direction of the electric field was changed by altering the HV connection with positive DC only—rather than changing the DC’s polarity.

### EFISH setup

**Figure 2:**
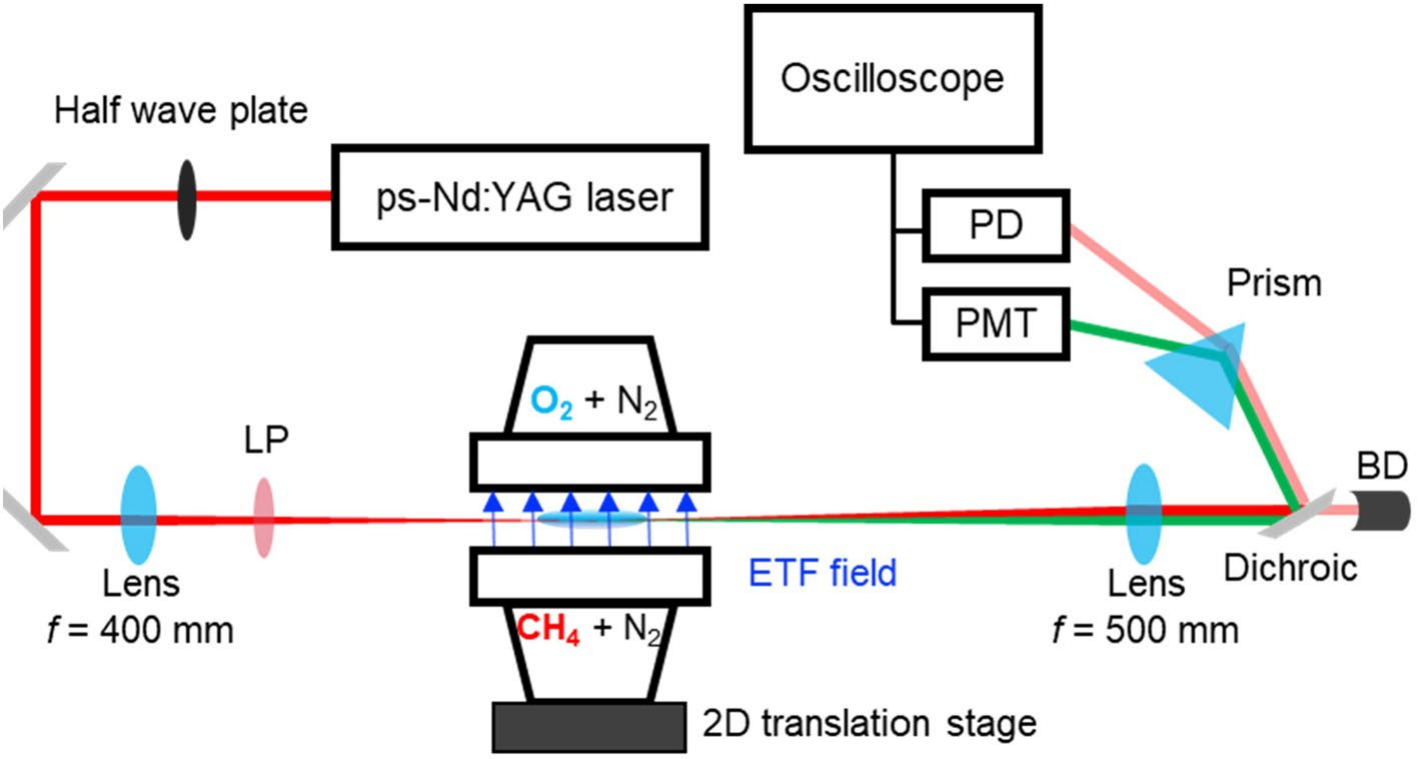
EFISH measurement system for counterflow flame (LP: long-pass filter, BD: beam dump, PD: photodiode, PMT: photomultiplier tube). Field direction in diagram represents ETF, as in Fig. 1.

The EFISH measurement system consisted of a picosecond Nd:YAG laser (Ekspla, PL2251A), lenses, dichroic mirrors, a half wave plate, a Long-Pass filter (LP), a prism, and detection devices, such as a PhotoDiode (PD, Thorlabs, DET025A) and a PhotoMultiplier Tube (PMT, Hamamatsu, H10721-01), as shown in Fig. 2. An incident beam (1064 nm) from the laser beam was polarized in *z*-direction through the half waveplate and focused on the center (the jet axis) of the burner, using a convex lens (focal length = 400 mm). The LP filter (> 800 nm), placed in the downstream of the convex lens, removed any parasitic second harmonic (532 nm) generated from the optical elements in the upstream. As the focused incident beam passed through the electric field, a Second Harmonic Signal (SHS, 532 nm) was generated from the incident beam. A second convex lens (focal length = 500 mm) collected both incident and SHS beam and collimated them again. Finally, a prism separated the incident and SHS beam spatially, and the PD and the PMT measured each intensity, respectively, in conjunction with the oscilloscope. A delay generator (BNC, Model 575) moderated the timing between the laser beam and the ns-HV switch to precisely measure the optical signal at a voltage condition of interest.

The burner setup was placed on a 2-D translational stage to align the incident beam with the center of the burner and move the measuring point along the *z*-axis vertically. The interval between measuring points was fixed at 0.1 mm. Due to interference caused by the electrodes’ surfaces, the incident beam could not probe the proximity to both electrodes (< 0.7 mm).

### Numerical method

To obtain numerical data, an OpenFOAM-based in-house CFD code was employed, which can simulate a flame with externally applied electric fields. The governing equations for the mass, momentum, species, and energy were as follows^30^:

1 $$\frac{\partial \rho }{\partial t}+\nabla \cdot (\rho \mathbf{u})=0,$$

2 $$\frac{\partial \rho \mathbf{u}}{\partial t}+\nabla \cdot \left(\rho \mathbf{u}\otimes \mathbf{u}\right)=-\nabla p\mathbf{I}+\nabla \cdot {\varvec{\uptau}}+\rho \mathbf{g}+{\mathbf{f}}_{\mathrm{e}},$$

3 $$\frac{\partial \rho {Y}_{k}}{\partial t}+\nabla \cdot \text{(}\rho \mathbf{u}{Y}_{k})=-\nabla \cdot {\mathbf{J}}_{k}+{\dot{\omega }}_{k},$$

4 $$\frac{\partial \rho {e}_{t}}{\partial t}+\nabla \cdot \left( \rho \mathbf{u}{e}_{t}\right)=\nabla \cdot \left[-\mathbf{q}+\left({\varvec{\uptau}}-p\mathbf{I}\right)\cdot \mathbf{u}\right]- \, \rho \sum_{k}^{M}{h}_{k}{\dot{\omega }}_{k}+\rho g\mathbf{u},$$

where *ρ*, $\mathbf{u}$, *p*, $\mathbf{I}$, and ${\varvec{\uptau}}$ are the density, velocity vector, pressure, identity tensor, and stress tensor, respectively. The specific governing equations for the present axisymmetric cylindrical coordinate can be found in SI. In the momentum equation (Eq. 2), $\rho \mathbf{g}$ is the gravity and ${\mathbf{f}}_{\mathrm{e}}$ is the electric body force due to space charges and the local electric field, which can be expressed as below, based on the Lorentz force:

5 $${\mathbf{f}}_{\mathrm{e}}=\sum_{k}^{M}{q}_{k}e\mathbf{E}{n}_{k},$$

where *M* is the total number of types of charged species; *q*_*k*_ and *n*_*k*_ are charge number and the number density of charged species of *k*, respectively; *e* is the elementary charge (*e* = 1.602 × 10^−19^ C). The electric field, **E**, is defined by the Gauss law as below:

6 $$\nabla \cdot \mathbf{E}=-{\nabla }^{2}V=\frac{\sum_{k}^{M}{q}_{k}e{n}_{k}}{{\varepsilon }_{0}},$$

where *V* is the electric potential, $\sum_{k}^{M}{q}_{k}{n}_{k}$ is the net charge density (*n*_c_), and *ε*_0_ (= 8.85 $\times$ 10^−12^ F/m) is the permittivity of free space. In the species equation (Eq. 3), *Y*_*k*_, **J**_*k*_, and ${\dot{\omega }}_{k}$ are the mass fraction, diffusion flux, and the net production rate of the species *k*. In the energy equation for a mixture containing *M* species (Eq. 4), *e*_*t*_ and **q** are the total energy and heat flux, respectively.

The diffusion flux of a neutral species is calculated based on Fick’s law as **J**_*k*_ =  − *ρD*_*k*_*Y*_*k*_*,* neglecting the Soret effect, where *D*_*k*_ is the diffusion coefficient of the species *k*. The Sutherland law was employed to obtain the diffusion coefficient for the neutral species^31^. The diffusion flux of a charged species must include the ambipolar diffusion due to the electric field, as shown below:

7 $${\mathbf{J}}_{k}=\rho {Y}_{k}{\mathbf{v}}_{k}=-\rho {D}_{k}{Y}_{k}+\frac{{q}_{k}}{\left|{q}_{k}\right|}\rho {Y}_{k}{\mu }_{k}\mathbf{E},$$

where *v*_*k*_ and *µ*_k_ are the diffusion velocity and mobility of the charged species *k*, respectively.

To obtain the transport coefficient of the electron and the ions, a numerical model, suggested by previous studies, was employed^26,27^. The binary mobility, *µ*_*ij*_, of an ion was calculated using Eq. (8)^26^, and the binary diffusivity, *D*_*ij*,_ was calculated using Einstein’s relation, as in Eq. (9)^26^:

8 $${\mu }_{ij}=\frac{3}{16}\frac{{q}_{i}e}{{\Omega }_{\mathrm{D}}(T){N}_{\mathrm{B}}}\sqrt{\frac{2\pi {N}_{\mathrm{A}}}{{k}_{B}T{m}_{ij}}},$$

9 $${D}_{ij}=\frac{{k}_{\mathrm{B}}T}{e}{\mu }_{ij},$$

where, *m*_*ij*_ is the reduced molar mass, *k*_B_ is the Boltzmann constant, *T* is the gas temperature, *N*_B_ is the background gas density, and *N*_A_ is the Avogadro number. The collision cross sections, *Ω*_D_, were obtained from the LXcat repository as a function of the electron temperature^28^. Following the assumption in the previous study^27^, we assumed the thermal equilibrium condition for all participating species including ions and electrons (*T* = *T*_ion_ = *T*_electron_); hence, the cross-section data obtained were based on gas temperature. For the mobility of electrons, Eq. (10) was used, as in^27^:

10 $${\mu }_{e}=\frac{2\gamma e}{3}\frac{1}{p{\widetilde{\sigma }}_{m}}\sqrt{\frac{{k}_{\mathrm{B}}T}{\uppi }},$$

where, *γ* = (2*e*/*m*_e_)^1/2^, *m*_*e*_ = 9.1094 × 10^−31^ kg, and ${\widetilde{\sigma }}_{m}={\sum }_{i}^{M}{X}_{i}{\Omega }_{Di}\left(T\right)$ is the mixture effective cross section for electron/neutral collisions, respectively. The diffusivity of electrons was also calculated using Eq. (9).

For the combustion kinetic mechanism, 40 neutral species were considered, reduced from Aramco 2.0 by maintaining the accuracy of a flame propagation speed, ignition delay, and the production of CH radicals. Eleven charged species were adopted including electrons, and the chemi-ionization mechanism was employed (consisting of 63 reactions), suggested by Belhi et al. and validated to reasonably predict voltage-current (V-I) relations in flat premixed flames^29^. The ion-mechanism could be considered as the most advanced one in the combustion community.

**Figure 3:**
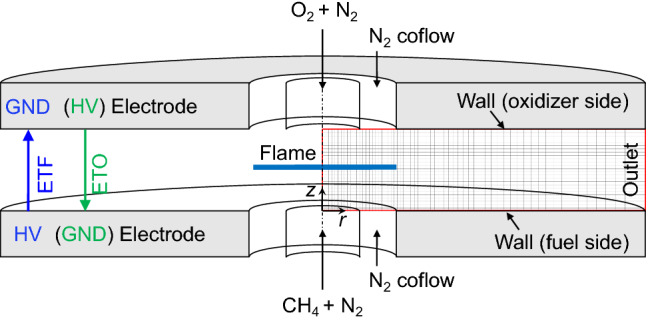
Schematic of counter-flow burner and computational geometry with boundary conditions.

We configured a 2D axisymmetric computational domain as shown in Fig. 3. A radial grid size in a flame zone (*r* < 10 mm) was maintained at 25 µm and gradually increased to 100 µm for *r* > 10 mm. A vertical grid size was uniformly maintained at 25 µm throughout the domain. The computational cost was 0.5 M core hours to achieve a converged solution using 860 CPU cores and 5-ns time step. The detailed grid- and time-independence test results can be found in Figs. S3 and S4 (SI)^32^. Table 1 shows the Boundary Conditions (BCs) for the simulation. Depending particularly on the direction of the electric field, the BCs for the charged species and electric potential at each electrode were applied differently.

**Table 1:** Boundary conditions for computational domain.

		Fuel side			Oxidizer side			Outlet
		Central jet	N_2_-sheath	Wall	Central jet	N_2_-sheath	Wall	
Momentum		*u* = 20 cm/s		*u* = 0	*u* = 20 cm/s		*u* = 0	Inlet/outlet
Neutral species		$Y_{{{\text{CH}}_{{4}} }}$ = 0.14 $Y_{{{\text{N}}_{{2}} }}$ = 0.86 *Y*_*k*_ = 0	$Y_{{{\text{N}}_{{2}} }}$ = 1.0 *Y*_*k*_ = 0	d*Y*_*k*_/d*t* = 0	$Y_{{{\text{O}}_{{4}} }}$ = 0.56 $Y_{{{\text{N}}_{{2}} }}$ = 0.44 *Y*_*k*_ = 0	$Y_{{{\text{N}}_{{2}} }}$ = 1.0 *Y*_*k*_ = 0	d*Y*_*k*_/d*t* = 0	
Charged species	ETO^a^	d*Y*^+^/d*t* = 0 and *Y*^–^ = 0			d*Y*^–^/d*t* = 0 and *Y*^+^ = 0			
	ETF^b^	d*Y*^–^/d*t* = 0 and *Y*^+^ = 0			d*Y*^+^/d*t* = 0 and *Y*^–^ = 0			
Electric Potential	ETO	*V* = *V*_a_			*V* = 0			d*V*/d*t* = 0
	ETF	*V* = 0			*V* = *V*_a_			d*V*/d*t* = 0

^a^ETO (electrons move through oxidizer stream): upward electric field.

^b^ETF (electrons move through fuel stream): downward electric field.

### Selection of experimental conditions

**Figure 4:**
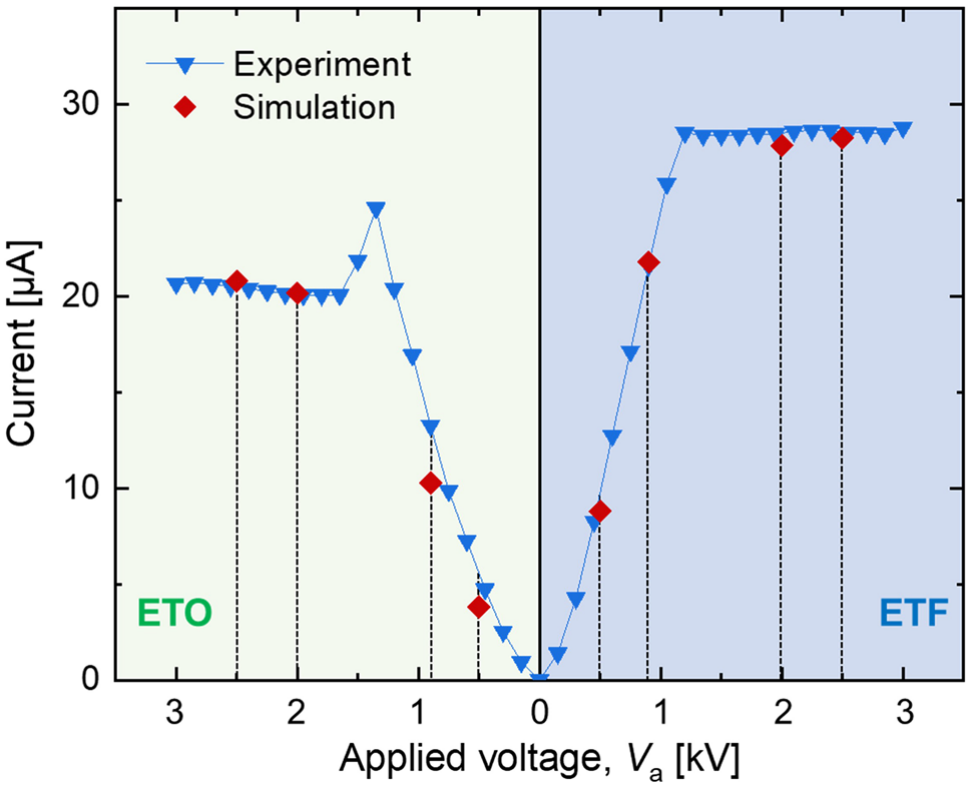
Measured and simulated current against applied voltage for tested methane nonpremixed flame. Vertical dashed lines indicate selected *V*_a_ conditions.

To determine experimental conditions, which can describe the characteristic regimes of the flame under the electric fields well, a preliminary experiment was conducted, measuring a V-I trend (shown in Fig. 4). In general, for both ETO and ETF cases, the sub-saturated regime was identified as parabolically increased current trends with increased applied voltage, while the constant currents characterized the saturated regime. This is a well-known phenomenon, and a physical mechanism has been explained clearly in literature^1,6,7^. In the case of ETO, the saturated current was lower than that of ETF, and the flame became unstable at around *V*_a_ = 1.5 kV (ETO), similar to the previous observation with nonpremixed propane flame^6^. In the sub-saturated regime, the rate of current increase with respect to *V*_a_ was higher in the case of ETF than in the ETO. This can be attributed to the lack of space charges in the fuel stream for ETF, since there are limited chances for electrons to form negative ions in collisions with CH_4_, pyrolyzed species, or N_2_^2,3^. This type of different electrical response caused by space charges, depending on the field direction, has been theoretically well described previously^7^.

In each direction of the electric field, four different applied voltages were chosen for both experimental and numerical conditions. Depicted with dashed vertical lines in Fig. 4, *V*_a_ = 0.5 and 0.9 kV and *V*_a_ = 2.0 and 2.5 kV represent the sub-saturated and saturated regime, respectively, for both ETO and ETF cases (see Table 2).

**Table 2:** Test conditions for both ETO and ETF field directions.

Regime in V-I trend	Applied voltage, *V*_a_ [kV]
Sub-saturated	0.5
	0.9
Saturated	2.0
	2.5

The numerically obtained currents for these 8 cases are also coplotted in Fig. 4. To match the experimentally obtained saturation current, the reaction rate coefficient of CH + O → CHO^+^  + e^−^ (initiation step for the chemi-ionization) was tuned from a literature value^3^ by 150%. This tuning method was also adopted in previous studies^25,29^.

## Measurement and calibration schemes

### EFISH measurement in a counterflow flame

When the laser beam is exposed to the electric field, the electric field induces the second harmonic signal, ${I}_{2\upomega }$, with a Gaussian incident beam profile, as follows:

11 $${I}_{2\upomega } \propto {\left[{\chi }^{\left(3\right)}\left(z\right) \cdot N\left(z\right)\cdot \mathbf{E}\left(z\right) \cdot {I}_{\upomega }\right]}^{2} \times {\left|{\int }_{0}^{L}\frac{\mathrm{exp}\left(i\cdot \Delta kx\right)}{1+{\left(\frac{x}{{z}_{\mathrm{R}}}\right)}^{2}}dx\right|}^{2},$$

where *χ*^(3)^ is the third order non-linear susceptibility, *N* is the number density of species, **E** is the electric field in *z* direction, *I*_ω_ is the intensity of the incident beam, *L* is the path length of the laser beam exposed to the electric field, ∆*k* is the wave vector mismatch between the incident and second harmonic wave, *z*_R_ is the Rayleigh range of a Gaussian beam, and *x* is the propagating direction of the incident beam^16^. Particularly, *χ*^(3)^ and *N* are strongly affected by gas composition and temperature, which vary significantly across a flame region.

In this regard, we suggested a calibration method of EFISH that could quantify the electric field without knowing *χ*^(3)^ and *N* in our previous study^20^. In the suggested method, Eq. (12) was deduced from Eq. (11) by assuming a uniform contribution of the path-length dependent term:

12 $$\sqrt{{S}_{\mathrm{EFISH}}} \equiv \frac{\sqrt{{I}_{2\omega }}}{{I}_{\omega }} \propto {\chi }^{\left(3\right)}\left(z\right) \cdot N\left(z\right)\cdot \mathbf{E}\left(z\right),$$

The total electric field, **E**_tot_, is the sum of the externally applied electric field, **E**_ext_, and the electric field formed by the space charge, **E**_sc_.

13 $${\mathbf{E}}_{\mathrm{tot}}= {\mathbf{E}}_{\mathrm{ext}}+ {\mathbf{E}}_{\mathrm{sc}},$$

Using the ns-HV switch, **E**_sc_ could be effectively preserved when the applied voltage was abruptly reduced. Note that **E**_ext_ on the flame could be reasonably assumed to be uniform due to the parallel configuration of the electrodes and the relatively large diameter of the electrode (80 mm) compared to the flame (~ 20 mm in diameter).

### Saturated regime

**Figure 5:**
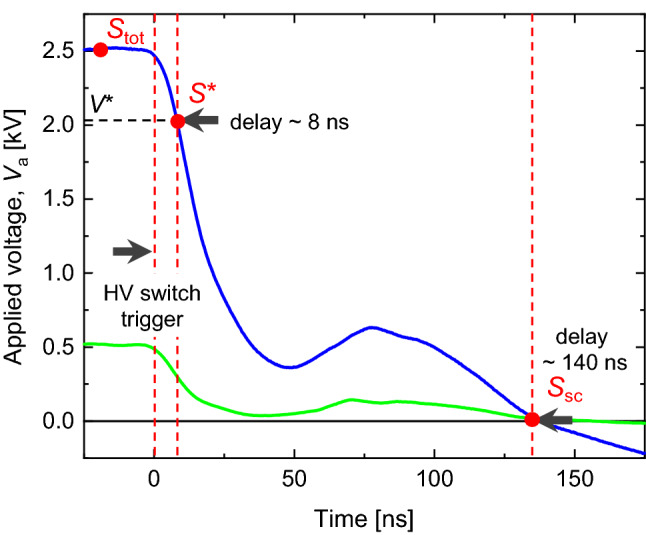
Voltage profile during the voltage-off using ns-HV switch and measurement points in time. The green and blue lines indicate the results for *V*_a_ = 0.5 and 2.5 kV cases, respectively.

For the saturated regime (**E**_ext_ $=$ 2.0 and 2.5 kV/cm), two EFISH signals were collected at each measuring point. One signal was with the intended *V*_a_, referred to as *S*_tot_, and the other signal (S*) was with an abruptly reduced applied voltage (V*), obtained at 8 ns after turning off the voltage using the ns-HV switch, as shown in Fig. 5. By doing this, it was possible to maintain the beam integration length (same as the electrode’s diameter) and **E**_sc_. Comparing *S*_tot_ and S*, a calibration constant, *c*_sat_(*z*) (explicitly including the information of *χ*^(3)^ and *N* at a measuring point), can be obtained as follows: the subtraction of those two EFISH signals in Eq. (12) yields

14 $$\sqrt{{S}_{\mathrm{tot}}}-\sqrt{{\mathrm{S}}^{*}}= {\chi }^{\left(3\right)}\left(z\right)\cdot N\left(z\right)\cdot \left({\mathbf{E}}_{\mathrm{tot}}-{\mathbf{E}}^{\boldsymbol{*}}\right).$$

By substituting **E**_tot_ and E* with **E**_ext_ (at *V*_a_) + **E**_sc_ and **E**_ext_ (at V*) + **E**_sc_, respectively, as in Eq. (13), the calibration constant can be obtained with the known Δ**E**_ext_ = **E**_ext_ (at *V*_a_) – **E**_ext_ (at V*) and measured EFISH signals as

15 $${c}_{\mathrm{sat}}\left(z\right)\equiv \frac{1}{{\chi }^{\left(3\right)}\left(z\right)\cdot N\left(z\right)} = \frac{{\Delta \mathbf{E}}_{\mathrm{ext}}}{\sqrt{{S}_{\mathrm{tot}}}- \sqrt{{\mathrm{S}}^{*}}} .$$

Note that Δ**E**_ext_ = 0.41 and 0.48 kV/cm were experimentally obtained at *V*_a_ = 2.0 and 2.5 kV, respectively.

Finally, the electric field can be deduced using the calibration constant and measured EFISH signal (*S*_tot_):

16 $${\mathbf{E}}_{\mathrm{tot}}\left(z\right)={c}_{\mathrm{sat}}(z)\cdot \sqrt{{S}_{\mathrm{tot}}} .$$

### Sub-saturated regime

In the sub-saturated regime however, measuring the EFISH signal (*S*_tot_ $$)$$ in the vicinity of the flame led to an inappropriate interpretation, since the local electric field in the flame is null caused by the electric field screening^1,7^. Although an anticipating electric field was null in this case, non-zero *S*_tot_ was detected due to that the beam was exposed to a finite electric field outside of the radial edge of the flame. Therefore, to obtain the null electric field properly, the vertical domain was divided into two regions, a near electrode region and a central region, for the calibration in the sub-saturated regime.

**Figure 6:**
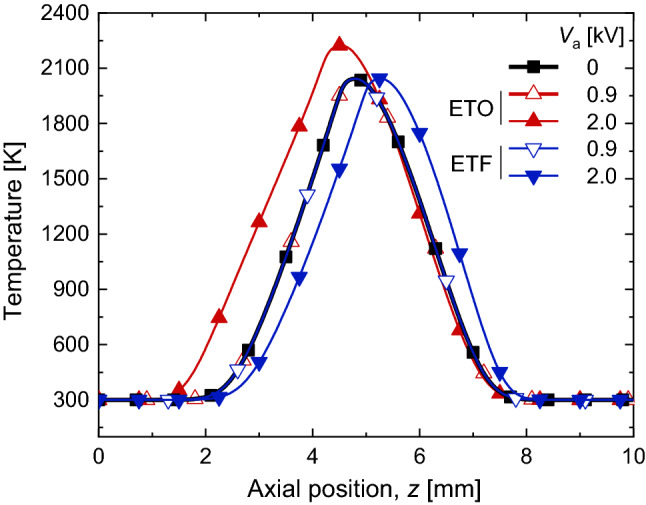
Numerically obtained temperature profiles along the center line for sub-saturated regime for various *V*_a_ with both ETO and ETF.

In the near electrode region, the local gas temperature and composition were minimally affected by the external electric field. Based on the numerical result shown in Fig. 6, the temperature in the near electrode region was maintained at 300 K for both saturated and sub-saturated regimes, regardless of the field direction. In addition, the local mixture compositions could also be reasonably maintained, since the ion density due to the electric field was negligible (smaller than 10^9^ cm^−3^^2^). Therefore, in this region (0–2 mm and 8–10 mm for ETF; 0–1.5 mm and 8–10 mm for ETO as shown in Fig. 6), the calibration constant, *c*_sat_(*z*), obtained for the saturated regime could be used. *S*_tot_ was measured at the intended *V*_a_, and **E**_tot_ was deduced from Eq. (16).

On the other hand, the EFISH signal (*S*_sc_) at *V*(*t*) = 0 (140 ns after turning off the voltage as in Fig. 5) from the intended *V*_a_ was measured in the central region (*z* = 2–8 mm for ETF; 1.5–8 mm for ETO). Because *S*_sc_ was purely originated from the field applied by the space charges only (**E**_sc_), **E**_tot_ by adding the uniform **E**_ext_ to **E**_sc_ (as in Eq. 13) could be reasonably determined, once *S*_sc_ was properly calibrated.

**Figure 7:**
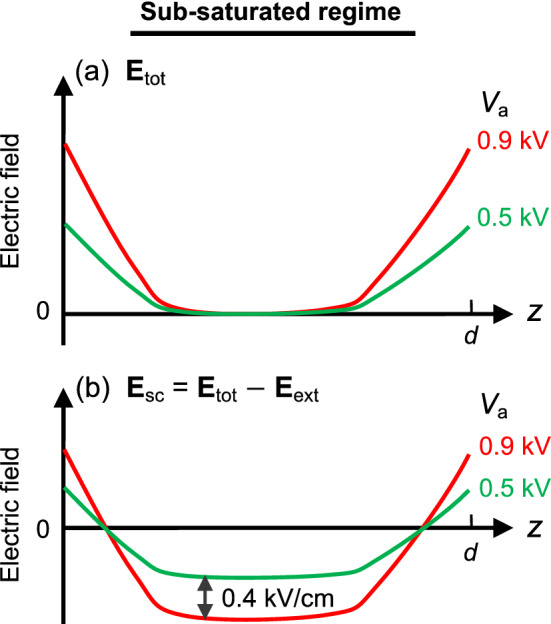
Schematic of the electric field for the sub-saturated regime: (**a**) total electric field, **E**_tot_, and (**b**) electric field due to space charges, **E**_sc_.

To calibrate *S*_sc_, we recalled that there must be a flat and null electric field (**E**_tot_(*z*) = 0) in a region surrounding the flame, due to the electric field screening effect^1,7,33^ in the sub-saturated regime. As shown schematically in Fig. 7a, there must be a region with null and flat **E**_tot_(*z*) for both *V*_a_ = 0.5 and 0.9 kV. The range of *z* for **E**_tot_(*z*) = 0, was unknown, but it had to be within the central region. **E**_sc_ was drawn schematically for both *V*_a_ by subtracting uniform **E**_ext_ = 0.5 and 0.9 kV/cm, respectively (Fig. 7b). It became clear that the difference between two flat electric fields in both **E**_sc_ must be same as the difference between two **E**_ext_ (0.9–0.5 = 0.4 kV/cm). Because the original temperature profile without *V*_a_ was maintained in the sub-saturated regime for both ETF and ETO cases (Fig. 6) and the number density of charged species was negligible, the calibration constant, *c*_sub_(*z*), for the flat region in the sub-saturated regime could be calculated by modifying Eq. (15):

17 $${c}_{\mathrm{sub}}\left(z\right)= \frac{0.4\mathrm{ kV}/\mathrm{cm}}{\sqrt{{S}_{\mathrm{sc}}{|}_{0.9}}- \sqrt{{S}_{\mathrm{sc}}{|}_{0.5}}},$$

where *S*_sc_|_0.5_ and *S*_sc_|_0.9_ indicate *S*_sc_ for *V*_a_ = 0.5 and 0.9 kV, respectively. **E**_sc_ was calculated as follows:

18 $${\mathbf{E}}_{\mathrm{sc}}\left(z\right)={c}_{\mathrm{sub}}(z)\cdot \sqrt{{S}_{\mathrm{sc}}} .$$

**E**_tot_ can be obtained by adding the known **E**_ext_ to **E**_sc_ (Eq. 13). It should be noted that we could not fully calibrate *S*_sc_, because Eq. (17) was valid only for the overlapped flat regions between two *V*_a_ cases.

**Figure 8:**
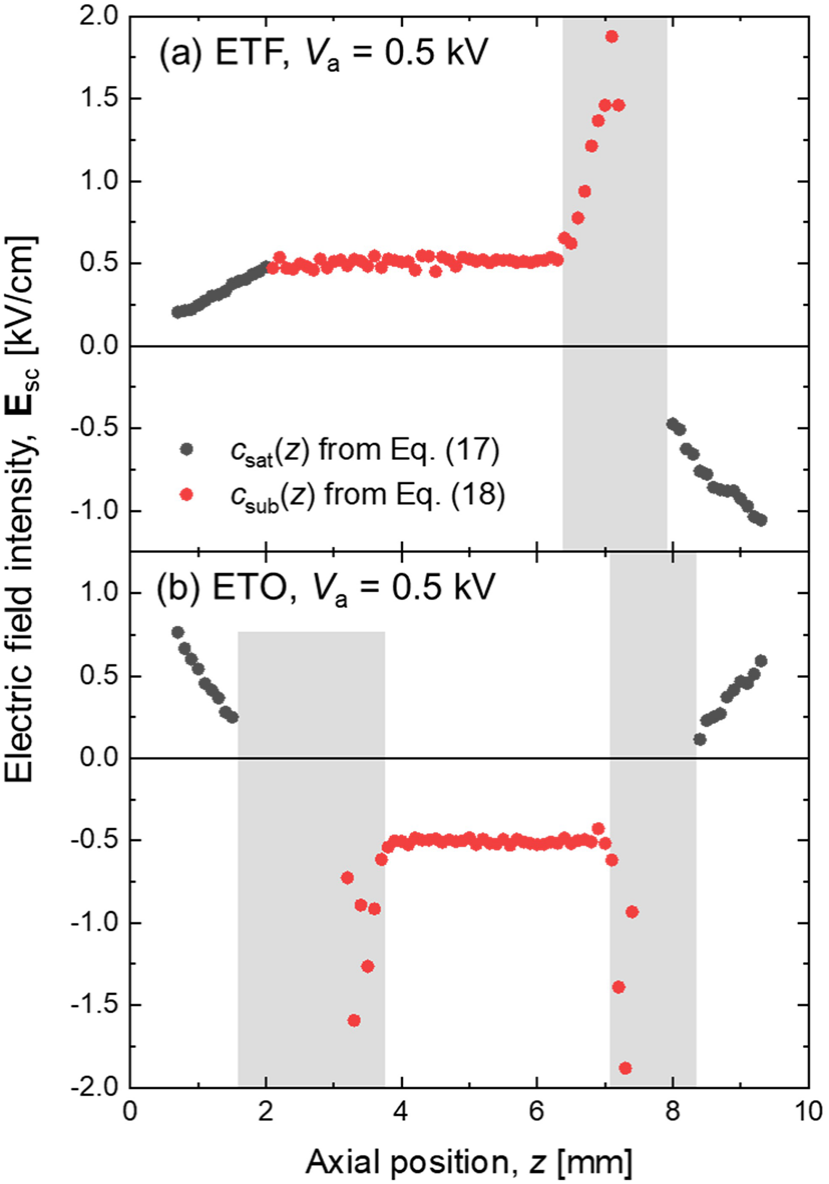
Calibrated electric field profiles of *V*_a_ = 0.5 kV cases for both (**a**) ETF and (**b**) ETO cases. Positive electric field means upward field direction.

The applicable range of *z* for *c*_sub_(*z*) could be determined, as we plotted the resulted **E**_sc_ for the entire *z* (0–10 mm). Figure 8 shows the resulted **E**_sc_ for both ETF (a) and ETO (b) cases at *V*_a_ = 0.5 kV. For the near electrode region, *c*_sat_(*z*) obtained from Eq. (15) was used to convert *S*_tot_ into **E**_tot_ first, and then **E**_sc_ was deduced by subtracting the known **E**_ext_ from **E**_tot_ (Eq. 13), whereas, for the central region, *c*_sub_(*z*) was used (Eq. 18). As *c*_sub_(*z*) was intrinsically valid only for the region of the flat electric field, the resulting **E**_sc_ in Fig. 8 shows the self-evident region of validity. It should be 2 < *z* < 6.4 mm and 3.8 < *z* < 6.9 mm for the ETF and ETO case, respectively. The continuous **E**_sc_ for the ETF case at *z* = 2 mm across the border between two regions (Fig. 8a) also supports the applicability of the proposed calibration method.

However, for the shaded areas in Fig. 8, the deduced **E**_sc_ using *c*_sub_(*z*) deviated significantly from the uniform electric fields. Because Δ**E**_sc_ (*z*) was no longer Δ**E**_ext_ = 0.4 kV/cm for the outside of the overlapped null electric fields, it was not appropriate to use *c*_sat_(*z*). Unfortunately, these shaded areas remain open for a future calibration.

## Results and discussion

**Figure 9:**
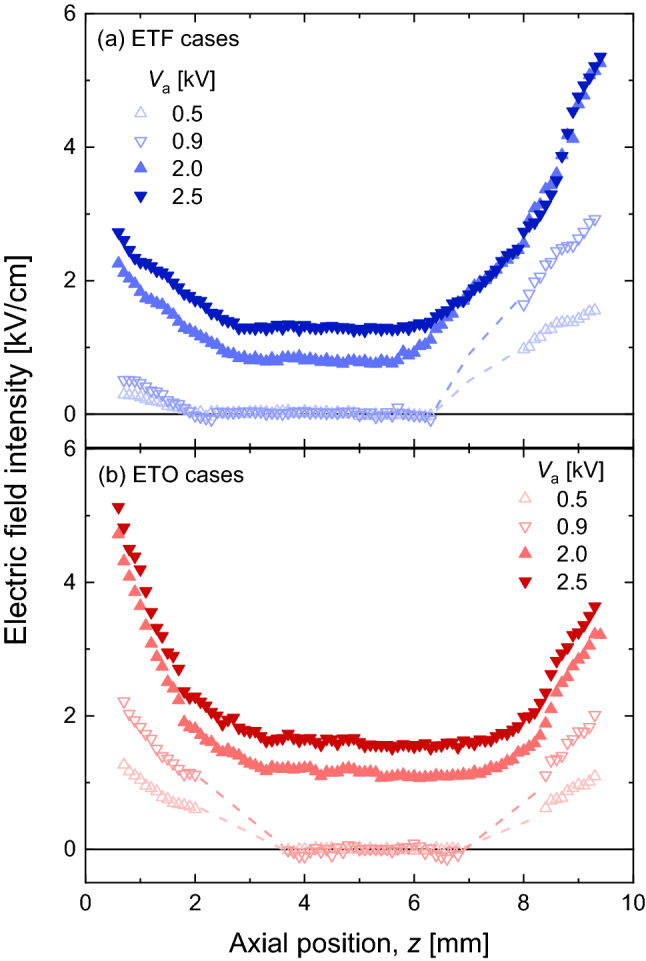
The resulted electric field profiles for all tested cases: (**a**) ETF and (**b**) ETO cases. (Hollow symbols for the sub-saturated regime; solid symbols for the saturated regime).

The measured electric field data for all tested conditions are illustrated in Fig. 9. In all cases, the electric fields were nearly flat in the central region with the flame inside, and the increased electric fields were observed towards both electrodes.

In the sub-saturated regime, the null electric fields due to the electric field screening effect by the ions and electrons in the flame zone were properly captured. As *V*_a_ increased, the electric field near the electrodes increased, while the null field in the central region remained (comparing the cases at 0.5 and 0.9 kV).

For the saturated regime, the overall electric field profiles seem to be lifted from the sub-saturated one. Although the electric fields were uniform in the central region, the null field cannot be observed anymore because there were no ions and electrons left in the flame zone (by the nature of the saturated regime) to shield the field from the external one. For further increased *V*_a_, the uniform electric field in the central region also increased (comparing the cases at 2.0 and 2.5 kV).

### Comparison for ETO cases

In the ETO cases, electrons readily create an O_2_^−^ via the electron attachment reaction^3^ because these move through the oxygen stream, while positive ions are evacuated through the fuel stream. As the previous one-dimensional theoretical study showed^7^, this effective electron transfer to heavier molecules resulted in increased negative space charges in the oxygen stream. Those space charges near both electrodes resulted in the augmented local electric field. Thus, for both saturated and sub-saturated regimes, the profile of the electric field showed a relatively symmetrical shape, demonstrating minimum field intensity near the flame region as shown in Fig. 9.

**Figure 10:**
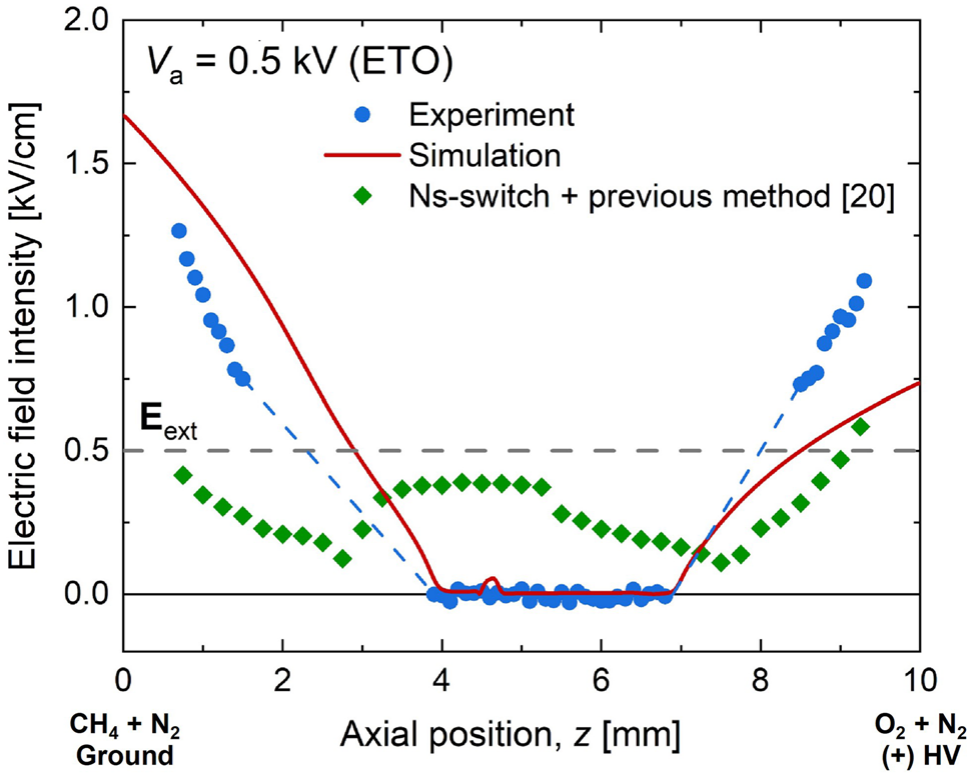
Measured and simulated electric fields for ETO at *V*_a_ = 0.5 kV (sub-saturated regime). For comparison, additional measurement using our previous method^20^ adopting ns-switch was coplotted.

For the sub-saturated regime at *V*_a_ = 0.5 kV (Fig. 10), the null electric field due to the electric field screening effect can be identified in 4 < *z* < 7 mm for both numerical and experimental results. The numerical result overpredicted the experimentally determined electric field in the fuel stream, while it underpredicted near the oxygen nozzle. Overall, both results agreed well with each other, and for the regions having no calibration method yet (1.5 < *z* < 4 mm and 7 < *z* < 8.5 mm), linear dashed lines were used to connect two regions for visibility.

The newly proposed measurement and calibration method in this study could be an accurate way to conduct an EFISH experiment. An extra experiment was performed adopting the previous method^20^ using the ns-switch. The previous method did not consider matching the beam integration length between *S*_tot_ and *S*_sc_ and should also bring significant uncertainty with the null electric field surrounded by a finite one. Unlike the numerical and the present experimental results, it showed a non-zero electric field in the central region (flame zone). It is also interesting to compare other result in a previous study using a different EFISH calibration method^22^. The resulted electric field in a similar counterflow geometry did not exhibit a null region even at the sub-saturated regime, demonstrating uniform one across the entire gap^22^, which was far from the trend of the present numerical result.

**Figure 11:**
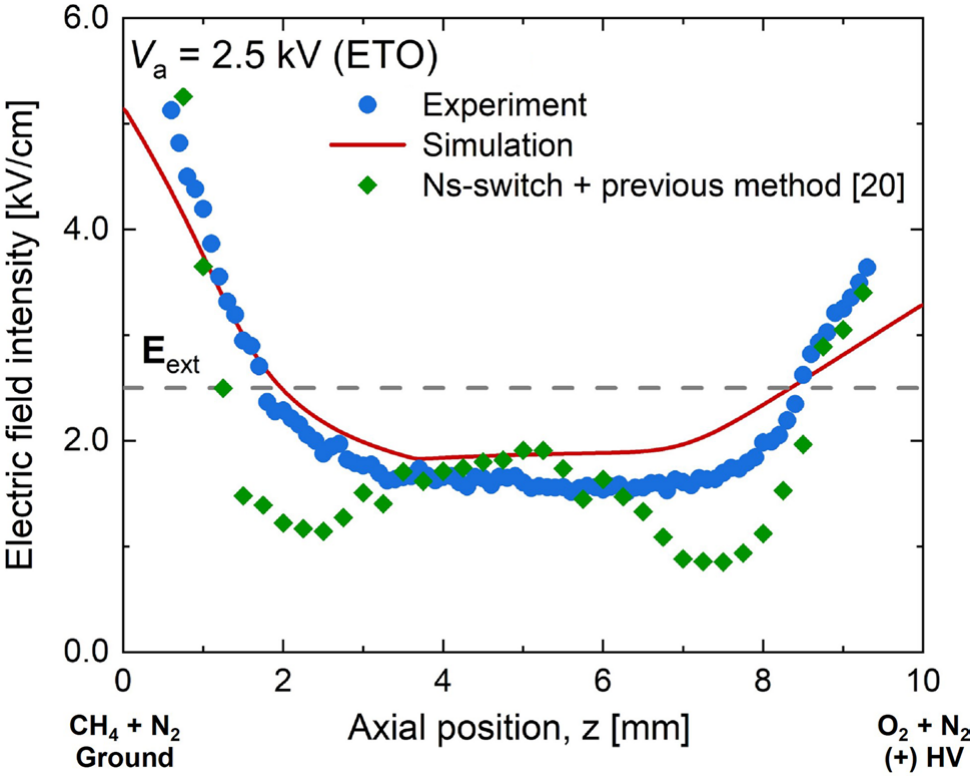
Measured and simulated electric fields for ETO at *V*_a_ = 2.5 kV (saturated regime). For comparison, additional measurement using our previous method^20^ adopting ns-switch was coplotted.

For the saturated regime (Fig. 11 at *V*_a_ = 2.5 kV), the measured and simulated electric fields also showed a good agreement. The simulation slightly overpredicted the electric field in and near the flame (central region) and underpredicted near both electrodes. An additional experiment with the previous method^20^ adopting the ns-switch did not show better agreement with both numerical and experimental results. This indicates that, although there was no null electric field in the flame region in the saturated regime, the influence of the integration length on the EFISH could not be insignificant, supporting the validity of the present method.

### Comparison for ETF cases

**Figure 12:**
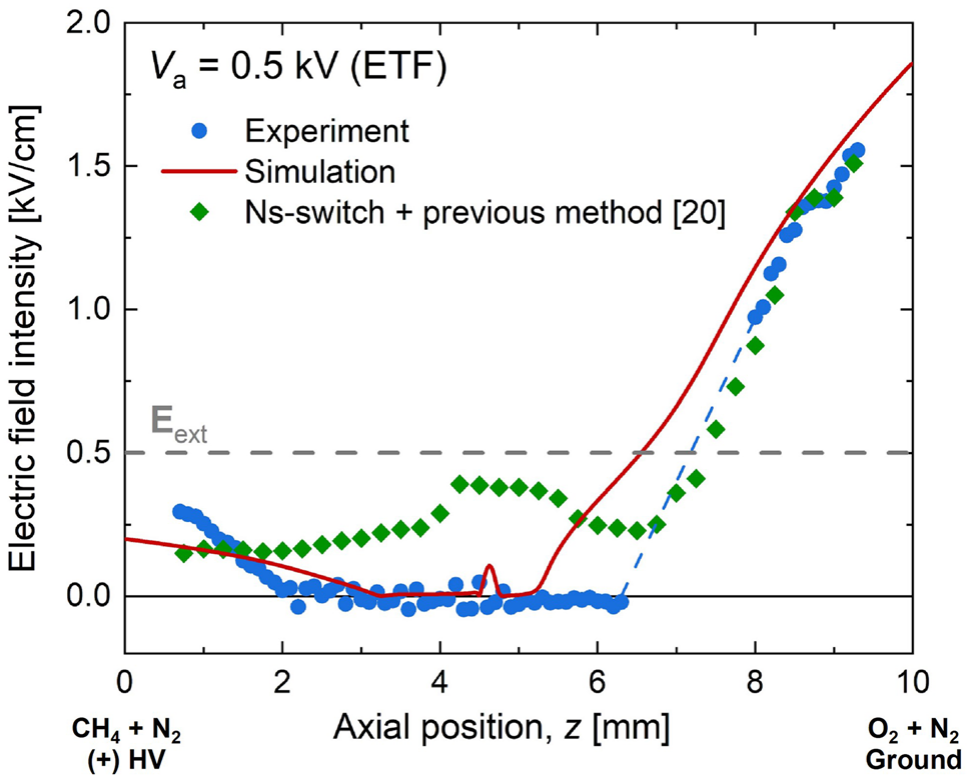
Measured and simulated electric fields for ETF at *V*_a_ = 0.5 kV (sub-saturated regime). For comparison, additional measurement using our previous method^20^ adopting ns-switch was coplotted.

In the ETF cases, we could also confirm that the present methods produced physically reasonable data as compared with the numerical result and the additional experimental data obtained using the previous method^20^ with the ns-switch. As shown in Fig. 12, the experiment with the previous method adopting the ns-switch could not capture the null electric field in the sub-saturated regime. The present method showed a qualitatively good agreement with the numerical result. In detail, the numerical result showed a near null electric field in a range of 3.5 < *z* < 5 mm, while the experimentally observed null electric field could be found in a larger domain (2 < *z* < 6.5 mm). In addition, the numerically obtained electric field near the ground electrode (oxygen stream) showed a good agreement with the experiment, but it significantly underpredicted the experiment near the HV electrode (fuel stream).

Both experimental and numerical results showed slightly increased electric fields near the anode (fuel stream). This implied that there must be negative space charges in the fuel stream. The numerically found composition of the space charge near the fuel outlet was CHO_3_^–^ (93%) and electrons (6.1%) although overall number density was extremely low as compared to that of H_3_O^+^ (99.9%) near the ground electrode. The numerical result also showed that H_3_O^+^ and electrons were two major charged species in the flame region. This indicated that there were no significant proton transfer reactions from H_3_O^+^ to others, as it moved through the oxygen stream. Also, considering no negative ions, originated from the non-oxidative fragments of the fuel in the ion-chemistry^3^, and the very low charge density in the numerical result, electron transfer to CHO_3_^–^ could be insignificant, resulting in fast evacuation of electrons from the system for the sub-saturated regime with ETF field direction.

**Figure 13:**
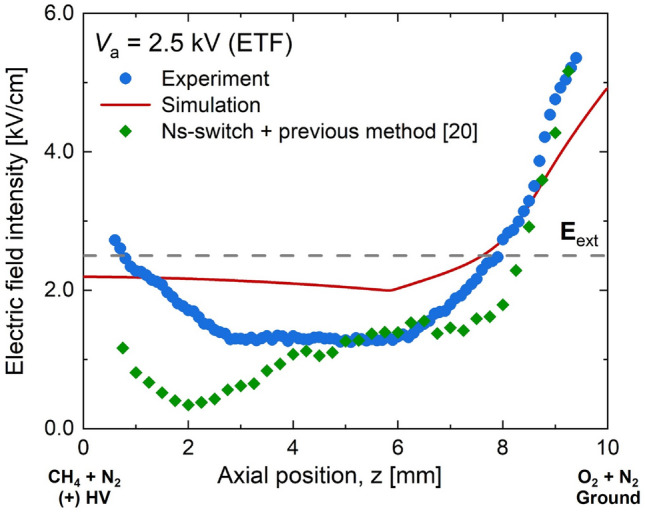
Measured and simulated electric fields for ETF at *V*_a_ = 2.5 kV (saturated regime). For comparison, additional measurement using our previous method^20^ adopting ns-switch was coplotted.

Unlike the previously compared cases, the biggest discrepancy between the experiment and the simulation was found in the ETF saturated regime (Fig. 13). Although the experimental and numerical results showed a good agreement for *z* > 7.5 mm, the profiles and values of both electric fields did not match well for *z* < 7.5 mm. Particularly, this discrepancy could be attributed to the negative space charges near the fuel outlet as mentioned previously, because the augmented electric field near the fuel stream could only be possible when there existed a significant pile of negative ions based on Gauss’s law. Considering the ion-chemistry adopted in the present study, electrons are expected to pass through the fuel stream, leaving a negligible footprint of ions due to the absence of O_2_. This resulted in a limited increase in the electric field toward the HV electrode in the simulation. Note that the additional experiment with the previous method^20^ adopting the ns-HV switch showed even worse profile.

As discussed above, in the ETF cases, electrons were expected to move through fragmented fuel species, with little chance to form negative ions, based on the well-known ion-chemistry^3^. Thus, the experimental result showing a significant level of negative charges in the fuel stream seems to be somewhat counterintuitive. In the numerical result, the most populated negative ion was CHO_3_^−^, demonstrating negligible concentration as compared to positive ions in the oxygen stream. To explain this experimentally obtained augmented electric field near the fuel outlet, we may need to revisit the used modeling and ion-chemistry. Considering the significant discrepancy between the experiment and numerical results was only found in the ETF cases, specifically due to negative space charges in the fuel stream, we think that the ion-chemistry needs to include carbonaceous ionic species such as C_2_H^−^, C_4_H^−^, and C_3_^−^. Goodings et al*.* found these carbonaceous negative ions in a methane-oxygen flame experimentally^34^. Therefore, the in-situ measurement of the ionic species in the presence of the external electric field can help to confirm the validity of the experimental findings and, thus, improve the ion-chemistry. A mass spectrometry will be used in a modified burner system by collecting samples at the center of nozzle exit.

## Conclusions

Electric fields in a counterflow nonpremixed flame with externally applied uniform electric fields were obtained experimentally and numerically. To quantify the electric field, a picosecond-laser based EFISH (Electric Field Induced Second Harmonic generation) measurement was performed. To overcome some known issues with the conventional EFISH method, we proposed new measurement and calibration schemes that were specific to the regime of the voltage-current (V-I) response in the flame. A multi-physics CFD simulation was also conducted, using an in-house OpenFOAM-based code with the state-of-the-art modeling and kinetic mechanisms to compare with the experimental results.

Motivated by our previous study^20^, an ns-high-voltage switch was successfully employed to effectively preserve the electric field due to space charges, while the applied voltage (*V*_a_) dropped abruptly. In the saturated regime, to obtain local calibration constants for EFISH, the integration length of the incident beam exposed to the electric field was maintained. However, for the sub-saturated regime, to circumvent the issue of measuring null electric field near the flame surrounded by finite electric field, EFISH signals with the space charges only were measured.

Overall, the measured electric fields agreed reasonably well with the numerical results for both field directions—Electrons move Through a Fuel stream (ETF) or Oxygen stream (ETO), respectively—in the sub-saturated regime. Specifically, the null electric field caused by the electric field screening (due to the flame’s ions and electrons for the sub-saturated regime) was successfully captured. For the saturated regime, the measured electric fields qualitatively agreed well with the numerical results in the ETO cases, whereas, in the ETF case, significantly different electric fields were found in the fuel stream. The EFISH measurement indicated that there must be a significant number of negative ions in the fuel stream for the ETF. The implemented ion-mechanism in simulation could not produce a noticeable number of negative ions for the ETF cases, since all negative ions are produced from O_2_^−^.

To further confirm the validity of both experiment and simulation in ETF saturated cases, the in-situ measurement of ionic species in the fuel stream should be a future study. Applying the present experimental and numerical methods to a canonical premixed flame would also be very informative.

## Supplementary Information

Supplementary Information.

## Data Availability

The experimental and numerical data presented in this study will be available upon request to the corresponding author (M.S.C.).
