# Supplementary material for: Electric fields in a counterflow nonpremixed flame: measurement and simulation
Source: Sci Rep. 2023 May 10;13:7622. doi: 10.1038/s41598-023-34769-6 (PMC10172352; doi:10.1038/s41598-023-34769-6)
Supplement: Supplementary file 1 — Supplementary Information. [file 41598_2023_34769_MOESM1_ESM.pdf]

Supplementary Information

Electric fields in a counterflow nonpremixed flame: Measurement and simulation

Jin Park[1], Jinwoo Son[1], Thomas D. Butterworth[2], Min Suk Cha[1]

[1] King Abdullah University of Science and Technology (KAUST), Clean Combustion Research Center (CCRC), Physical Science and Engineering Division (PSE), Thuwal 23955, Saudi Arabia
[2] Brightsite Plasmalab, Circular Chemical Engineering, Maastricht University, Brightlands Chemelot Campus, Urmonderbaan 22, Geleen, 6167 RD, The Netherlanbds

Governing equations in an axisymmetric cylindrical coordinate

Continuity equation

$$\frac{\partial \rho}{\partial t}+\frac{1}{r}\frac{\partial r\rho u_r}{\partial r}+\frac{\partial \rho u_z}{\partial z}=0$$

Momentum equation

$$\frac{\partial \rho u_r}{\partial t}+\frac{1}{r}\frac{\partial r\rho u_r^2}{\partial r}+\frac{\partial \rho u_r u_z}{\partial z}=-\frac{\partial p}{\partial r}+\frac{1}{r}\frac{\partial r\tau_{rr}}{\partial r}+\frac{\partial \tau_{rz}}{\partial z}+F_r$$

$$\frac{\partial \rho u_z}{\partial t}+\frac{1}{r}\frac{\partial r\rho u_r u_z}{\partial r}+\frac{\partial \rho u_z^2}{\partial z}=-\frac{\partial p}{\partial z}+\frac{1}{r}\frac{\partial r\tau_{rz}}{\partial r}+\frac{\partial \tau_{zz}}{\partial z}+\rho g_z+F_z$$

Species equation

$$\frac{\partial \rho Y_k}{\partial t}+\frac{1}{r}\frac{\partial r\rho u_r Y_k}{\partial r}+\frac{\partial \rho u_z Y_k}{\partial z}=-\left(\frac{1}{r}\frac{\partial rJ_{k,r}}{\partial r}+\frac{\partial J_{k,z}}{\partial z}\right)+\dot{\omega}_k$$

Energy equation

$$\frac{\partial \rho e_t}{\partial t} + \frac{1}{r}\frac{\partial r\rho u_r e_t}{\partial r} + \frac{\partial \rho u_z e_t}{\partial z}$$

$$= -\left(\frac{1}{r}\frac{\partial rQ_r}{\partial r} + \frac{\partial Q_z}{\partial z}\right) - \left(\frac{1}{r}\frac{\partial r u_r p}{\partial r} + \frac{\partial u_z p}{\partial z}\right)$$

$$+ \left(\frac{2}{r}\frac{\partial r\tau_{rr}u_r}{\partial r} + \frac{1}{r}\frac{\partial r\tau_{rz}u_z}{\partial r} + \frac{1}{r}\frac{\partial r\tau_{rz}u_r}{\partial r} + 2\frac{\partial \tau_{zz}u_z}{\partial z} + 2\frac{\partial \tau_{rz}u_z}{\partial z}\right)$$

$$- \rho\sum_{k}^{M} h_k\dot{\omega}_k + \rho g_z u_z$$

where the stress tensor, τ_{ij} is:

$$\tau_{zz} = \mu\left(2\frac{\partial u_z}{\partial z} - \frac{2}{3}\left(\frac{\partial u_r}{\partial r} + \frac{\partial u_z}{\partial z} + \frac{u_r}{r}\right)\right),$$

$$\tau_{zr} = \tau_{rz} = \mu\left(\frac{\partial u_r}{\partial z} + \frac{\partial u_z}{\partial r}\right),$$

$$\tau_{rr} = \mu\left(2\frac{\partial u_r}{\partial r} - \frac{2}{3}\left(\frac{\partial u_r}{\partial r} + \frac{\partial u_z}{\partial z} + \frac{u_r}{r}\right)\right).$$

In this study, the viscosity(μ) was obtained by the Sutherland transport model as below:

$$\mu = \frac{A_s\sqrt{T}}{1 + T_s/T},$$

where A_s and T_s are the Sutherland coefficient. The coefficients have been released by UMIST and NIST.

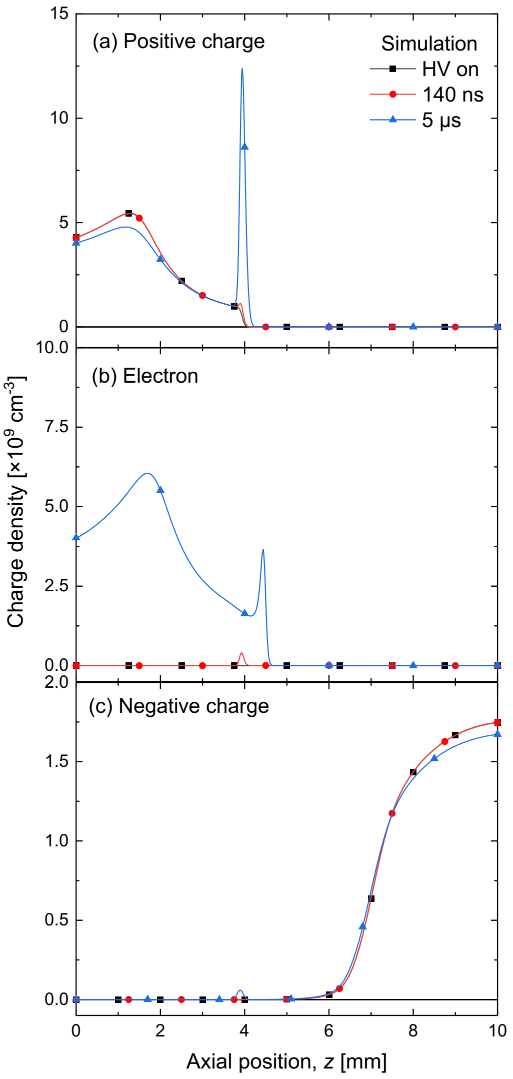

Fig. S1. The ns-HV switch can 'freeze' the motion of charged species effectively, since the charged species profiles are not much affected by the voltage-off duration of 140 ns. Numerical results show the charged species profiles along the jet axis, demonstrating the effect of voltage-off duration for the ETO case at $V_a = 2.5$ kV: (a) positive ions, (b) electrons, and (c) negative ions. At 140 ns (red in the figures) after turning off V_a, the positive ions displayed a nearly identical profile with that of steady HV case before turning off the voltage; in the case after 5 μs (blue in the figures), a peak appeared near the flame location due to the accumulation of generated ions for 5 μs. The number density of electrons was three orders of magnitude smaller than those of ions due to the difference in mobilities, and the electron profile did not change significantly after 140 ns. However, 5 μs seemed to be sufficient for the generated electrons to move toward the pile of positive ions, neutralizing the static field formed by the positive ions. This could alter the static field significantly. The negative ion profile did not appear to be significantly affected by the time duration until 5 μs.

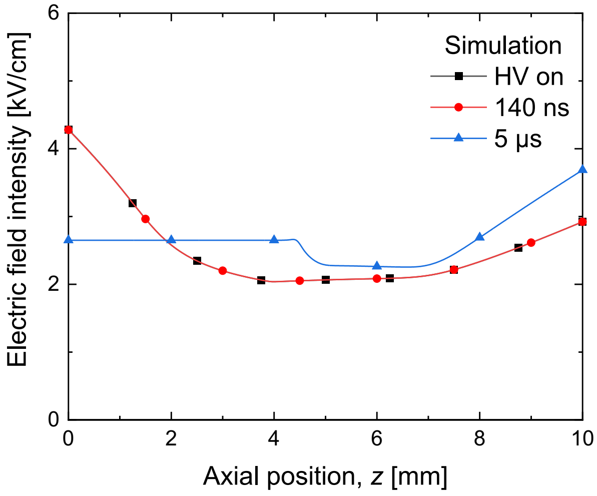

Fig. S2. The ns-HV switch can maintain the electric field effectively during the voltage-off duration of 140 ns. Numerically obtained electric field shows that the 140 ns duration of the voltage-off does not influence the overall electric field for ETO at V_a = 2.5 kV. Electric fields for cases after 140 ns and 5 μs were reconstructed using Eq. 13 (in the manuscript) to compare with $\mathbf{E}_{on}$ before turning off the voltage. This figure confirms that the original $\mathbf{E}_{on}$ before turning off the voltage and the reconstructed $\mathbf{E}_{on}$ after 140 ns were nearly identical. After 5 μs, on the other hand, $\mathbf{E}_{on}$ was disturbed by the change in charge distribution as shown in Fig. S1.

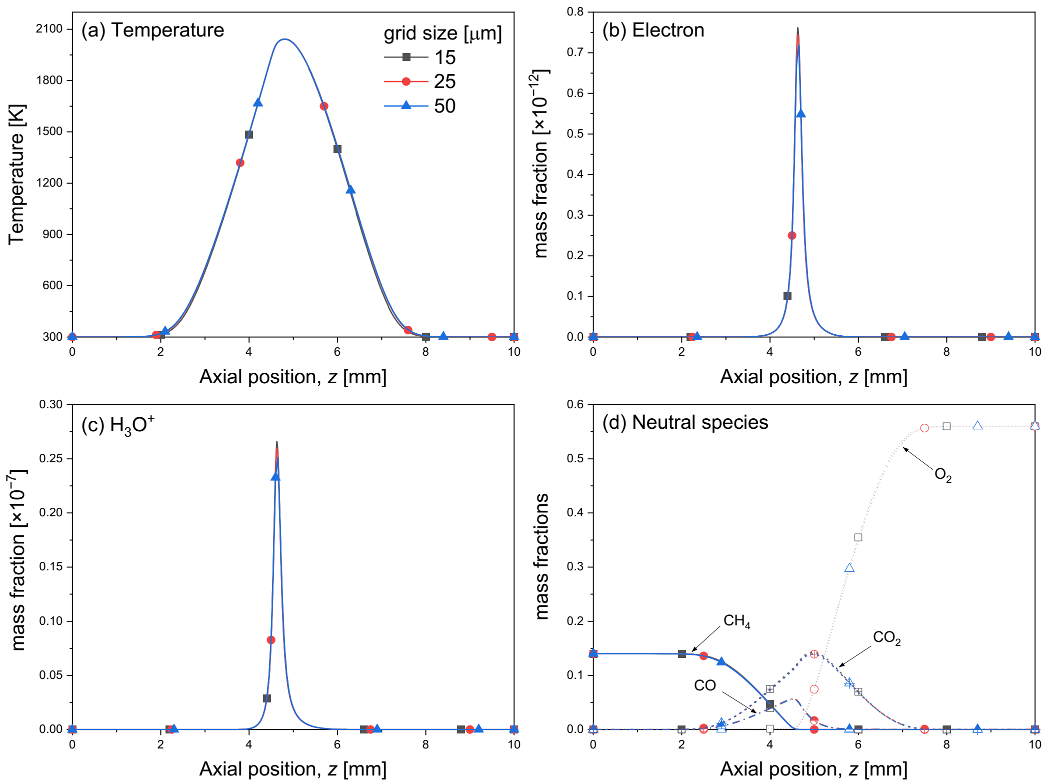

Fig. S3. Grid-independence test was conducted for three different gird sizes (15, 25, and 50 μm), and the results were compared in terms of (a) temperature and the mass fractions of (b) electron, (c) H_3O^+, and (d) selected neutral species (CH_4, O_2, CO, and CO_2). The selected neutral species and the temperature show negligible differences among three tested grid systems; while, both peak mass fractions of the electron and H_3O^+ show 2 and 6 % deviation at the grid size of 25 and 50 μm, respectively, as compared to those at the 15 μm grid system, demonstrating well agreed axial profiles with each other.

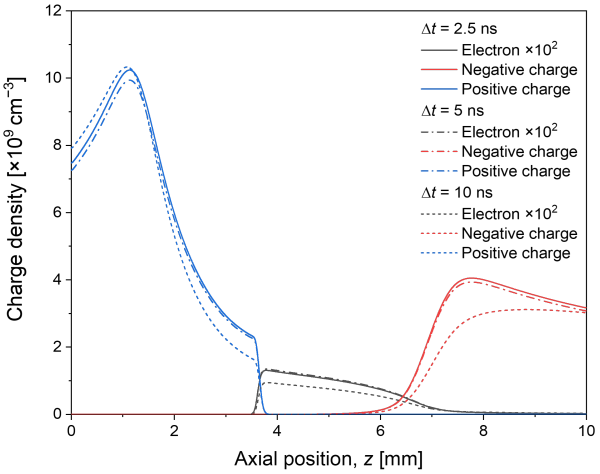

Fig. S4. Time-independence test was conducted using various time steps (Δt = 2.5, 5, and 10 ns) in the ETO case at V_a = 2 kV. Temperature and neutral species were not influenced by the tested range of Δt. Meantime, charged particles (electron, positive ions, and negative ions) show significant deviation at Δt = 10 ns. Reasonably agreed axial profiles are observed between two cases at Δt = 2.5 and 5 ns.
